# Diabetes and cancer: two diseases with obesity as a common risk factor

**DOI:** 10.1111/dom.12124

**Published:** 2013-06-12

**Authors:** S K Garg, H Maurer, K Reed, R Selagamsetty

**Affiliations:** 1 Barbara Davis Center for Diabetes, University of Colorado DenverAurora, CO, USA; 2 Diabetes Technology and TherapeuticsNew Rochelle, NY, USA; 3 Medicine and Pediatrics, University of Colorado DenverAurora, CO, USA; 4 Boston UniversityBoston, MA, USA

**Keywords:** antidiabetic drug, diabetes complications, diabetes mellitus, type 2 diabetes

## Abstract

There is a growing body of evidence to support a connection between diabetes (predominantly type 2), obesity and cancer. Multiple meta-analyses of epidemiological data show that people with diabetes are at increased risk of developing many different types of cancers, along with an increased risk of cancer mortality. Several pathophysiological mechanisms for this relationship have been postulated, including insulin resistance and hyperinsulinaemia, enhanced inflammatory processes, dysregulation of sex hormone production and hyperglycaemia. In addition to these potential mechanisms, a number of common risk factors, including obesity, may be behind the association between diabetes and cancer. Indeed, obesity is associated with an increased risk of cancer and diabetes. Abdominal adiposity has been shown to play a role in creating a systemic pro-inflammatory environment, which could result in the development of both diabetes and cancer. Here, we examine the relationship between diabetes, obesity and cancer, and investigate the potential underlying causes of increased cancer risk in individuals with diabetes. Current treatment recommendations for reducing the overall disease burden are also explored and possible areas for future research are considered.

## Introduction

Worldwide, cancer is the 2nd and diabetes the 12th leading cause of death 1. There is a growing evidence base to support a connection between diabetes, predominantly type 2 diabetes mellitus (T2DM), and certain types of cancer 2. In fact, meta-analyses have revealed T2DM to be an independent risk factor for the development of several different types of cancer 2. As yet, few studies have explored a relationship between type 1 diabetes mellitus and cancer 3.

The connection between diabetes and cancer was first postulated more than 75 years ago. Although these two conditions share many common risk factors, there continues to be a lack of understanding of the biological relationship between them, 2–3 which poses a challenge for patient care. Current thinking suggests that the relationship may not be entirely attributable to the direct effects of diabetes, such as hyperglycaemia 3,4. Instead, it may be that diabetes is a marker of altered cancer risk due to changes in underlying metabolic conditions, such as insulin resistance or hyperinsulinaemia 3.

Alternatively, the relationship may be due, wholly or in part, to the sharing of common predisposing conditions, such as obesity 3. In fact, both obesity and T2DM have been shown to be independently associated with an increased risk of cancer and mortality 5.

Unravelling the pathology behind the link between these diseases may be further complicated by the multidrug therapy required for the treatment of diabetes, obesity and cancer 3. There is even some evidence to suggest that established glucose-lowering therapies play a role in cancer development. Several observational studies in people with diabetes have shown a higher risk of cancer or cancer death with certain antidiabetic agents 6–9. However, some of the evidence is conflicting, and the non-randomized nature of these studies means that potential for selection bias to confound results is high. Furthermore, the relative contributions of increased risk due to one diabetes medication versus another cannot be determined from these studies 3. Recently presented prospective trials and database analyses have reported no increase in cancer with insulin glargine treatment 10–14.

Individually, the health consequences and economic costs of diabetes, obesity and cancer are high, and their impact is exacerbated when all three conditions co-present. In order to address some of the mounting concerns regarding the management of diabetes and cancer, the American Diabetes Association and American Cancer Society have published a consensus report providing recommendations for clinical care 3.

The aims of this review article are to further examine the association between diabetes, obesity and cancer, discuss the potential underlying causes and explore the current treatment recommendations for reducing the overall burden associated with these conditions.

## Evidence for a Link between Diabetes and Certain Types of Cancer

Numerous clinical studies have suggested a link between diabetes and a variety of different types of cancer, including breast 15, colorectal 16–17, endometrial 18–19 and pancreatic 20,21 cancers. Multiple meta-analyses of case–control and prospective cohort studies have confirmed that T2DM is an independent risk factor for the development of several different types of cancer including non-Hodgkin lymphoma (NHL) and cancers of the bladder, breast, colorectum, endometrium, liver and pancreas (Table1) 23–38. Larsson et al. conducted three separate meta-analyses of case–control and cohort studies investigating the risk of breast, bladder and colorectal cancers in association with T2DM (Table1). The authors reported modest associations between T2DM and both breast and bladder cancers, while a strong association was reported for colorectal cancer and associated mortality (relative risk 1.26, 95% confidence interval [CI] 1.05, 1.50) 23,24. A positive association between T2DM and NHL has also been observed (Table1) 30–31. As altered immune function and chronic inflammation are implicated in the pathogenesis of NHL, the underlying low-grade, systemic, pro-inflammatory state associated with T2DM may, therefore, contribute to the pathogenesis of NHL 31.

**Table 1:** Diabetes as a risk factor for cancer (summary of meta-analyses) 2

		Case–control studies		Prospective cohort studies	
Authors	Tumour type	n	RR (95% CI)	n	RR (95% CI)
Larsson et al. 23	Bladder	7	1.4 (1.0–1.8)	3	1.4 (1.2–1.7)
Larsson et al. 24	Breast	5	1.2 (1.1–1.3)	15	1.2 (1.1–1.3)
Wolf et al. 25	Breast	4	1.1 (1.0–1.3)	6	1.3 (1.2–1.3)
Larsson et al. 26	Colorectal	6	1.4 (1.2–1.5)	9	1.3 (1.2–1.4)
Friberg et al. 28	Endometrium	13	2.2 (1.8–2.7)	3	1.6 (1.2–2.2)
El-Serag et al. 29	HCC	13	2.5 (1.9–3.2)	12	2.5 (1.9–3.2)
Chao et al. 31	NHL	10	1.2 (1.0–1.4)	3	1.8 (1.3–2.5)
Mirri et al. 30	NHL	11	1.1 (0.9–1.3)	5	1.4 (1.1–1.9)
Everhart et al. 32	Pancreatic	11	1.8 (1.1–2.7)	9	2.6 (1.6–4.1)
Huxley et al. 33	Pancreatic	17	1.9 (1.5–2.5)	19	1.7 (1.6–1.9)
Bonovas et al. 34	Prostate	5	0.9 (0.7–1.2)	9	0.9 (0.9–1.0)
Kasper et al. 35	Prostate	7	0.9 (0.7–1.1)	12	0.8 (0.7–0.9)
Bansal et al. 38	Prostate	16	0.85 (0.74–0.96)	29	0.87 (0.80–0.94)

CI, confidence interval; HCC, hepatocellular carcinoma; NHL, non-Hodgkin lymphoma; RR, pooled relative risk.

Adapted with permission from Ref. 2.

Owing to the co-morbidities frequently associated with diabetes, as well as the complex nature of T2DM itself, each site-specific cancer needs to be evaluated individually in order to differentiate between risk factors associated with cancers in general and those that influence specific cancers 36.

### Diabetes and Prostate Cancer

A negative association has been shown between diabetes and the risk of prostate cancer (Table1), with this negative association increasing with increased duration of diabetes 34–39. This reduced risk was not seen in all studies, with a significant increased risk for prostate cancer seen in Asian populations 38–39. It has been suggested that the increased risk in these populations could be the result of different distributions of *AR*, *SRD5A2* and *VDR* genotypes which are associated with prostate cancer risk 38–40. Two large cohort studies found that men with diabetes are at higher risk for advanced prostate cancer at diagnosis 41–42. This higher risk could be related to the lower levels of testosterone and prostate-specific antigen (PSA) observed in men with diabetes, which results in a lower likelihood of PSA screening identifying early prostate cancer 41–43. Levels of PSA have been shown to be lowest in people who have had diabetes for a long duration; however, studies have not found a clear link between decreased PSA and decreased prostate cancer risk 44. The mechanism by which diabetes causes reduced PSA levels is also currently unknown 39.

Another possible biological mechanism linking diabetes with a protective effect on prostate cancer risk is the link between hyperinsulinaemia and prostate cell growth. Insulin is positively associated with the growth of both normal and cancerous prostate cells, and, therefore, decreased insulin production, as observed in people with diabetes, may inhibit cell growth 34. Hypoinsulinaemia may also suppress prostate cancer indirectly. Hypoinsulinaemia triggers a cascade of events which leads to an increase in the level of plasma insulin-like growth factor (IGF)-1 44. Elevated IGF-1 levels are seen as a risk factor for prostate cancer 45–46. However, in a study looking at the association between prostate cancer and diet, it was found that long-term exposure to a diet high in refined carbohydrates (i.e. one which elicits a high insulin response and reduced IGF-1 levels) did not decrease prostate cancer incidence as one would have expected, suggesting that other biological mechanisms may potentially be at work 47. Owing to their critical role in both prostate growth and prostate cancer development, and the fact that their levels differ according to diabetes status, androgens have been suggested to play a role in driving this inverse relationship between diabetes and prostate cancer 39. A pooled analysis that included 18 studies (3886 men with prostate cancer and 6438 controls) found no association between risk of prostate cancer and serum concentrations of eight sex hormones (including testosterone, dihydrotestosterone and estradiol), whilst there was a modest inverse association with sex hormone-binding globulin (p = 0.01) 48. Kasper et al. found that both testosterone and sex hormone-binding globulin levels increased significantly with increasing diabetes duration (p = 0.02 and 0.002, respectively) 49. At the same time, they observed that the ratio of testosterone to sex hormone-binding globulin decreased, which suggested that levels of bioavailable testosterone were reduced 49. Low levels of testosterone and sex hormone-binding globulin have also been shown to be predictive factors for the development of both metabolic syndrome and diabetes in middle-aged men 39–50. This suggests that the interaction between diabetes, sex-hormone levels and prostate cancer is complex, with increased levels of sex hormone-binding globulin being a possible factor in the reduced incidence of prostate cancer.

### Diabetes and Pancreatic Cancer

There is an added level of complexity in the association between diabetes and pancreatic cancer, as both diseases involve the same organ, with studies suggesting that diabetes could be both an early manifestation of pancreatic cancer and an aetiological factor 51,52. This is because pancreatic cancer can cause abnormal glucose metabolism, and risk factors for pancreatic cancer include obesity, chronic pancreatitis and diabetes 54. A meta-analysis of cohort studies by Ben et al. found that diabetes is associated with a mean 1.94 greater risk of pancreatic cancer 52. The highest risk of pancreatic cancer (relative risk: 5.38; 95% CI: 3.49–8.30, p < 0.001) was observed in those diagnosed with diabetes for less than a year, supporting the hypothesis that diabetes, at least in some cases, may be induced by pancreatic cancer and thus may be an early indicator of this cancer. The relative risk of developing pancreatic cancer decreased in those people who had been diagnosed with diabetes for at least 10 years (relative risk: 1.47; 95% CI: 0.94–2.31) 52. Likewise, results from two case–control studies including 688 pancreatic cancer cases and 2204 controls reported that the odds ratios (ORs) for pancreatic cancer were more pronounced among those diagnosed with diabetes in the previous 2 years (OR: 5.17; 95% CI: 2.71–9.87) than among those with diabetes diagnosed more than 2 years ago (OR: 2.35; 95% CI: 1.70–3.26). The ORs remained significantly elevated 2–4 years and 5–9 years since diagnosis of diabetes, after which a non-significant 20% increased risk for pancreatic cancer was observed 55.

In addition, it has been found that individuals who develop exocrine pancreatic cancer tend to have moderate increases in glycated haemoglobin (HbA1c) levels, relatively independent of obesity and insulin resistance 56. Indeed, a study by Pannala et al. reported that 85% of people with pancreatic cancer have either impaired glucose tolerance or diabetes mellitus 51. Furthermore, diabetes was predominantly of new onset (<2-year duration) in 74% of those with diabetes and pancreatic cancer. Following pancreaticoduodenectomy, diabetes resolved in 57% of patients with new-onset diabetes, whilst its prevalence was unchanged in those with long-standing diabetes 51–57. A retrospective analysis of clinical data from 331 pancreatic patients by Huang et al. confirmed these findings. Results from this analysis reported that most patients were diagnosed with diabetes mellitus either concomitantly with pancreatic cancer (39.0%) or within 6 months before cancer diagnosis (6.9%); only 7.9% of patients were diagnosed with diabetes 24 months before pancreatic cancer diagnosis 53. Because subjects with new-onset diabetes over the age of 50 years have an eightfold increased risk of pancreatic cancer, new-onset diabetes with weight loss may be a potential indicator of pancreatic cancer in this population 57. It has been suggested that diabetes secondary to benign or malignant disease of the exocrine pancreas is type 3c (secondary) diabetes to distinguish its aetiology 54.

## Diabetes and Cancer Mortality

Mortality rates in diabetes, obesity and cancer populations are high, with both T2DM and obesity being independently associated with an increased risk of cancer-related mortality 5–64. Evidence from a large, prospective, US cohort has shown diabetes to be an independent predictor of mortality associated with cancer of the colon and the pancreas in both men and women, with breast cancer in women, and with cancer of the liver and bladder in men 58. Furthermore, a meta-analysis of 23 studies has shown that preexisting diabetes may be a mortality risk factor [hazard ratio (HR): 1.41; 95% CI: 1.28–1.55] in people with newly diagnosed cancer compared with normoglycaemic individuals across all types of cancer 61. In particular, a significant increase in mortality risk was observed for endometrial, breast and colorectal cancers 2–61. A recent analysis of 17 cohorts involved in the Diabetes Epidemiology: Collaborative Analysis of Diagnostic Criteria in Europe (DECODE) study has further corroborated an increased cancer risk in people with diabetes, with differences in cancer risk observed between men and women 60 (Table2).

**Table 2:** Cancer mortality in men and women with diabetes 60

Type of cancer	Men	Women
All cancer		
Mortality	8.52	5.04
HR (95% CI)	1.44 (1.21–1.70)	1.35 (1.08–1.68)
Stomach		
Mortality	1.74	0.24
HR (95% CI)	1.84 (1.25–2.71)	0.48 (0.19–1.21)
Liver		
Mortality	0.73	0.43
HR (95% CI)	5.16 (2.56–10.41)	6.37 (2.18–18.62)
Pancreas		
Mortality	0.77	0.62
HR (95% CI)	1.67 (0.94–2.97)	2.13 (1.09–4.16)
Bronchus/lung		
Mortality	1.21	0.52
HR (95% CI)	0.88 (0.58–1.35)	0.93 (0.48–1.81)
Prostate		
Mortality	1.31	—
HR (95% CI)	1.30 (0.84–2.01)	—
Breast		
Mortality	—	0.81
HR (95% CI)	—	1.65 (0.93–2.93)
Kidney/bladder		
Mortality	0.53	0.33
HR (95% CI)	1.20 (0.61–2.37)	1.97 (0.75–5.15)

Data for all-cause diabetes expressed as per 1000 person-years; HR adjusted for study cohort, age, body mass index, systolic blood pressure, cholesterol and smoking status. CI, confidence interval; HR, hazard ratio.

Adapted with permission from Ref 60, Table 3.

The Health, Eating, Activity, and Lifestyle (HEAL) study of women diagnosed with stage I-IIIA breast cancer found that increasing insulin resistance and beta-cell dysfunction were associated with reduced breast cancer survival (HR: 1.12; 95% CI: 1.05–1.20) and reduced all-cause survival (HR: 1.09; 95% CI: 1.02–1.15) 65. In a study of people with early-stage breast cancer, the risk of all-cause mortality was found to be twice as high in women with HbA1c ≥ 7% (≥53 mmol/mol) compared with women with HbA1c <6.5% (<47.5 mmol/mol; HR: 2.35; 95% CI: 1.56–3.54) 66. Interestingly, another study has linked current tamoxifen therapy with an increased incidence of diabetes in older breast cancer survivors, suggesting that tamoxifen treatment exacerbates an underlying risk of diabetes in susceptible women 67.

There are a number of possible reasons for this elevated mortality. These include presentation of more advanced disease at diagnosis in people with diabetes 68, which may be compounded by increased tumour cell proliferation and metastases in a physiological environment of hyperinsulinaemia and hyperglycaemia 69. Owing to screening issues with diabetes, cancer is often not diagnosed as early in people with diabetes as in those without diabetes; for example, women with diabetes were less likely to undergo mammography or colorectal screening, and diabetes increased the likelihood of a breast cancer diagnosis at a late stage of malignancy by 19% 70–71. Also, obese women are reported to have lower breast and cervical cancer screening rates 63–72. Alternatively, the presence of cancer may result in suboptimal treatment of diabetes and diabetes-related co-morbidities 61, while a diabetes diagnosis and/or diabetes-related co-morbidities could influence the selection of cancer treatment. Analysis of registry data from 58 498 people with cancer in The Netherlands showed a general trend towards less aggressive cancer treatment in people with diabetes (n = 5555) than in those without diabetes (n = 52 943; p < 0.05) 62. There is also some evidence to suggest that people with diabetes may be less responsive to chemotherapy than people without diabetes 73. Lastly, individuals with diabetes are more likely to have a cancer recurrence 74 and to have an increased risk of complications (e.g. infections) resulting in poorer outcome 75. Mortality risk has also been observed to increase in postoperative cancer patients who also have diabetes 76.

## Biological Link between Diabetes and Cancer

**Figure 1:**
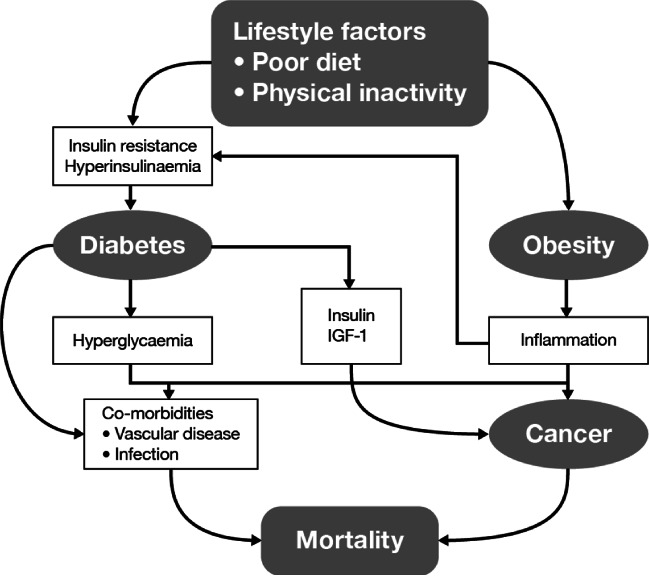
Interrelationship between pathological mechanisms and modifiable and non-modifiable risk factors involved in diabetes, obesity and cancer. IGF, insulin-like growth factor.

Several diabetes-related pathophysiological mechanisms, including hyperinsulinaemia, hyperglycaemia and inflammation, have been implicated in increasing cancer risk via their influence on neoplastic processes (figure 1) 4.

Insulin resistance, hyperinsulinaemia (either endogenous due to insulin resistance or exogenous due to administered insulin or insulin secretagogues) and elevated levels of IGF-1 reduce apoptosis and increase cell proliferation in target cells, leading to tumour development 36–79. Insulin itself is also known to have mitogenic properties 80, and, in particular, both the liver and the pancreas are exposed to a high level of endogenously produced insulin.

The effects of insulin and hyperinsulinaemia on tumorigenesis are thought to be mediated by the insulin receptor, which is expressed in both normal tissues and tumours 81–82. Activation of insulin receptor signalling pathways leads to proliferative and antiapoptotic events 82. Dysregulation of these signalling pathways during insulin resistance and hyperinsulinaemia can result in enhanced cell proliferation 83, and in cancer cells, this could enhance disease progression. IGF-1 and its receptor have also been implicated in the development of cancer by activating pathways of cell survival and proliferation 84. Insulin inhibits the production of IGF binding protein (IGFBP)-1 (and possibly IGFBP-2), leading to an increase in the levels of ‘free’ IGF-1, that is, the active form of the growth factor 85. This ‘free’ form of IGF-1 is then available to bind with IGF-1 receptors, which, like insulin receptors, are expressed in high numbers by cancer tissues 86. This binding initiates a signalling pathway within the cancer cells which favours tumour growth, activating mitogenesis and inhibiting apoptosis 87.

Production of the sex hormone oestrogen can also be modulated by insulin and hyperinsulinaemia, and this is thought to play a role in tumorigenesis 2. Indeed, it has been postulated that several mechanisms may underlie the association between breast cancer and diabetes, including activation of the insulin pathway, activation of the IGF pathway and altered regulation of sex hormones 88.

The production of pro-inflammatory cytokines, such as interleukin (IL)-6 and tumour necrosis factor (TNF)-*α*, is also thought to mediate an increase in the dysregulation of metabolic pathways leading to insulin resistance, which in turn leads to an increase in insulin levels, thus further increasing the inflammatory response 89. This is important because adipose tissue is regarded as a paracrine organ that secretes pro-inflammatory mediators 90. Diabetes is usually associated with a low-grade pro-inflammatory state, and this could be a key factor in encouraging tumorigenesis 91.

Elevated fasting serum glucose levels have been shown to be an independent risk factor for certain cancers, and the risk increases with rising glucose levels, as does cancer-related mortality 92. Hyperglycaemia has been shown to confer resistance to chemotherapy on breast cancer cells, as well as reduced overall survival 93–94, but appears to confer no growth advantage; given the molecular heterogeneity of cancer cells, a possible growth advantage of hyperglycaemia should not be ruled out 4. A study by Turturro et al. in the MDA-MB-231 cell line showed that the expression of the oxidative stress gene TXNIP is regulated by glucose level which in turn regulates reactive oxygen species levels, which can stimulate mitogenesis of cancer cells 95. Overall, however, the data suggest that insulin receptor activation is more integral to the development of cancer than hyperglycaemia 3.

### Links between Cancer Chemotherapy and Hyperglycaemia/Diabetes

Hyperglycaemia may impact on the outcome of chemotherapy for cancer 95–98. Experiments have shown that glucose increases the cytotoxicity of 5-fluorouracil 99, with a case series in seven people with diabetes suggesting that the degree of toxicity was directly related to the severity of hyperglycaemia 97. *In vitro* studies involving breast cancer cell lines have shown that hyperglycaemia directly impacts these cells 95–96. Results from a study that investigated the effect of hyperglycaemia on the cytotoxic effects of carboplatin and 5-fluorouracil in MCF-7 cells reported that a hyperglycaemic state increased the toxicity of the drugs by approximately 30% and reduced their IC_50_ by 1.5- and 1.3-fold, respectively 96. This increased cytotoxicity was seen to be potentiated by reduced P-glycoprotein expression, and possibly by increased reactive oxygen species levels. An explanation for this is that the reduced P-glycoprotein level increases accumulation and retention of the chemotherapeutic agent 96. Cancer chemotherapy may also result in hyperglycaemia, with drug-induced diabetes having been reported following treatment with a number of agents including 5-fluorouracil 100, glucocorticoids 101, androgen-deprivation therapy 41 and carboplatin/paclitaxel 102. These drugs may also worsen preexisting diabetes 103.

## Biological Link between Obesity and Cancer

### Clinical Studies

Obesity is a major confounding factor in studies of diabetes and cancer as the risk of developing T2DM increases with growing body mass 104. Clinical evidence has shown that increased measures of obesity, such as body mass index (BMI) and waist circumference, are associated with increased prevalence of certain cancers, such as pancreatic and prostate cancers (Table3) 105–109. The European Prospective Investigation into Cancer and Nutrition (EPIC; ∼520000 participants) has shown a link between increasing grades (magnitudes) of obesity and certain cancers 110–113. Studies also suggest that obesity can lead to inferior treatment outcomes and a poorer response to treatment, both chemo- and radiotherapy 114,115.

**Table 3:** Risk ratio for cancer per 5 kg/m^2^ higher body mass index 109

Cancer type	Men (RR [95% CI])	Women (RR [95% CI])	Suggested causal mechanism
Oesophageal adenocarcinoma	1.52 (1.33–1.74)*	1.51 (1.31–1.74)*	Reflux oesophagitis and chronic irritation
Thyroid	1.33 (1.04–1.70)†	1.14 (1.06–1.23)‡	Unknown
Colon	1.24 (1.20–1.28)*	1.09 (1.05–1.13)*	Insulin
Renal	1.24 (1.15–1.34)*	1.34 (1.25–1.43)*	Hypertension is one factor
Liver	1.24 (0.95–1.62)	1.07 (0.55–2.08)	Fatty liver cirrhosis
Malignant melanoma	1.17 (1.05–1.30)‡	0.69 (0.92–1.01)	Unknown
Multiple myeloma	1.11 (1.05–1.18)*	1.11 (1.07–1.15)*	Inflammatory pathways—IL-6
Rectum	1.09 (1.06–1.12)*	1.02 (1.00–1.05)	Unknown
Gallbladder	1.09 (0.99–1.21)	1.59 (1.02–2.47)†	Chronic secretion—gallstones and irritation
Leukemia	1.08 (1.02–1.14)‡	1.17 (1.04–1.32)†	Unknown
Pancreas	1.07 (0.93–1.23)	1.12 (1.02–1.22)‡	Possibly insulin pathways
Non-Hodgkin's lymphoma	1.06 (1.03–1.09)*	1.07 (1.00–1.14)	Inflammatory pathways—IL-6
Prostate	1.03 (1.00–1.07)	—	Unknown
Lung	0.76 (0.70–0.83)*	0.80 (0.66–0.97) ‡	People who smoke are more likely to be lean leading to bias and this cancer is caused by smoking
Oesophageal squamous	0.71 (0.60–0.85)*	0.57 (0.47–0.69)*	People who smoke are more likely to be lean leading to bias and this cancer is caused by smoking
Endometrium	—	1.59 (1.50–1.68)*	Endogenous oestrogen
Breast (postmenopausal)	—	1.12 (1.08–1.16)‡	Endogenous oestrogen
Breast (premenopausal)	—	0.92 (0.88–0.97)†	Irregular menstrual cycles, hormones

RR, risk ratio; CI, confidence interval.

* p < 0.0001;

† p < 0.01;

‡ p < 0.05.

* Biased to null because this includes predominantly low-grade lesions.

Adapted with permission from Ref. 109 Tables 1 and 2.

Body fat distribution can also be a marker of future risk, with abdominal adiposity being a potential mechanism of cancer development owing to increased levels of free fatty acids and cytokines, leading to insulin resistance and increased IGF availability 117. It has been reported that, after adjustment for BMI, there is an almost linear association between waist circumference and risk of death from colon cancer 64. Mortality risk has also been observed to increase in obese individuals with T2DM 118. Hormones are involved in the pathogenesis of obesity as well as in that of T2DM and cancer, as outlined previously. Elevated levels of endogenous oestrogen are thought to be linked to insulin resistance and hyperinsulinaemia as a direct result of inhibition of insulin receptor function 119.

Observations of increased risk of premenopausal breast cancer in obese individuals have been linked to altered regulation of inflammatory mediators and adipokine levels 120.

### Mechanism of Action

Several mechanisms have been proposed for obesity-induced cancers, including abnormalities in adipokine secretion, including hyperleptinaemia, by central adipose tissue, which plays a role in various stages of obesity-induced carcinogenesis 121–124. Diet is one of the main determinants of a person's body composition and plays an important role in obesity 122. The chronic state of over-nutrition associated with obesity may also be a factor, leading to the production of reactive oxygen species, which stimulate cancer cell mitogenesis 125. There is also the possibility that carcinogenic compounds within foods consumed could have more of an effect owing to the increased diet 122. Additionally, the inflammatory effects on the body that obesity causes may promote cancer progression and survival 126. Systemic inflammatory responses, such as increased production of TNF-*α*, IL-6, IL-1, C-reactive protein, plasminogen activator inhibitor-1 and fibrinogen, and tissue-specific hormone activity mediated through the activation of nuclear factor kappa-light-chain-enhancer of activated B cells and peroxisome proliferator-activated receptor gamma pathways appear to be mechanistically linked with obesity 127. Weight loss has been shown to lead to a decrease in the production of these inflammatory markers 128. Other health issues related to obesity can also result in higher risk of cancer, for example, the increased incidence of gastro-oesophageal reflux and Barrett's oesophagus in obese people increases the risk for oesophageal adenocarcinoma 109–122.

Obese people have also been shown to have poorer outcomes from treatment. For example, obese people undergoing surgical resection for colorectal cancer have been reported to have significantly longer operating times for laparoscopic surgery, as well as a greater likelihood of conversion from laparoscopic to open surgery 129–133. In some instances, the effectiveness of radiotherapy is reduced as there can be difficulties positioning obese patients and adjusting the volume of radiation needed for treatment 114–116. Many chemotherapeutic agents have a narrow therapeutic index, which can result in very obese people being underdosed owing to concerns that the large doses that are calculated based upon body weight will cause excess toxicity 109. However, studies have not shown an increase in chemotherapy-related toxicity in obese people treated at full intensity, suggesting that obese individuals are underdosed, thereby reducing the efficacy of the chemotherapy 109,115. Therefore, optimal dosing strategies for chemotherapeutic agents in obese people with cancer need to be defined 109.

## Current Diabetes Treatments May Play a Role in Cancer Development

Current diabetes treatments have been implicated in the development of cancer. Consequently, cancer risk may now play a part in the risk–benefit analysis that a physician undertakes when prescribing diabetes medication. The potential increased risk of cancer associated with diabetes medications may be a result of direct or indirect effects on insulin resistance and levels of circulating insulin or other mechanisms 135–138.

### Metformin

**Figure 2:**
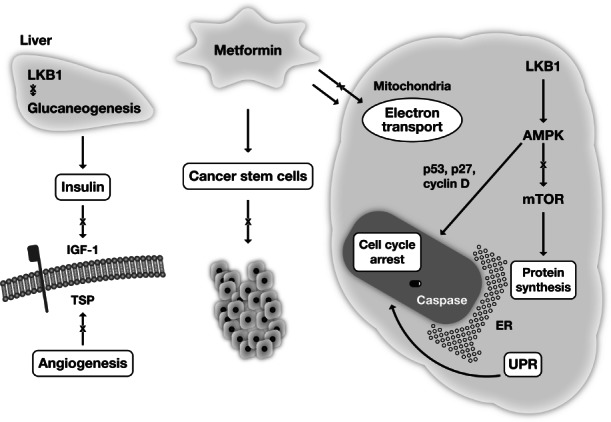
Possible mechanism by which metformin may be able to inhibit cancer cell growth. AMPK, adenosine monophosphate-activated protein kinase; ER, endoplasmic reticulum; IGF, insulin-like growth factor; LKB1, liver kinase B1; mTOR, mammalian target of rapamycin; TSP, thrombospondin; UPR, unfolded protein response. Adapted with permission from Ref. 144.

Metformin, the most commonly used therapy in people with T2DM 139, may have a protective effect against cancer, possibly because of a reduction in glucose and insulin levels or other mechanisms including the effects on adenosine monophosphate-activated protein kinase (AMPK) signalling pathways 8–143. In preclinical studies, metformin has been shown to inhibit cell proliferation, reduce colony formation and cause partial arrest in cancer cell lines 2. Other *in vitro* research has also shown that metformin may selectively kill cancer stem cells and enhance the effectiveness of breast cancer treatment regimens 3. Many new studies are investigating the possible mechanisms of the antineoplastic effects of metformin, and these have recently been eloquently summarized by Kourelis et al. (figure 2) 144. After a review of *in vitro* and *in vivo* studies, Kourelis et al. postulated that there are at least seven mechanisms that could account for the anticancer effects of metformin. These include: (i) activation of the liver kinase B1/AMPK pathway, in which metformin activates the AMPK pathways that inhibit the mammalian target of rapamycin pathway via phosphorylation, stabilizing *TSC2*, a tumour suppressor gene 145; (ii) induction of cell cycle arrest and/or apoptosis; (iii) inhibition of protein synthesis; (iv) reduction in circulating insulin levels; (v) inhibition of the unfolded protein response; (vi) activation of the immune system; and (vii) eradication of cancer stem cells (figure 2) 144.

A recent meta-analysis of 12 randomized controlled trials which compared metformin with other glucose-lowering therapies or placebo/usual care found no significant difference between the groups in terms of cancer risk 146. However, cancer data were absent from five of the trials investigated and the comparators were not homogeneous. In a recent large-scale study of the association between metformin use and the incidence of breast cancer in a T2DM population, there was about a 20% reduction in the rate of incident invasive breast cancer among women with T2DM using metformin compared with both non-metformin users and other antidiabetic medication users 147. Another study found that metformin had a statistically significant negative association with the development of pancreatic cancer 148.

The protective effect of metformin on cancer seen in epidemiological studies is so strong that metformin is now being investigated as an adjuvant therapy for cancer in three ongoing prospective trials. The Effect of Metformin on Breast Cancer Metabolism Study (NCT01266486) is investigating biomarkers that indicate a change in biological response in S6 kinase, 4E binding protein 1 and AMPK over a 2-week period. This study in metformin-naïve women with breast cancer aims to assess any metformin-induced phosphorylation of S6 kinase, 4E binding protein 1 and AMPK via immunohistochemical analysis, as well as investigating whether metformin could be used as a cancer therapy 144. The Metformin Hydrochloride as First-Line Therapy in Treating Patients With Locally Advanced or Metastatic Prostate Cancer Study (NCT01243385) is investigating whether metformin can be used to treat early prostate cancer in metformin-naïve men. The trial is also using immunohistochemistry to analyze the phosphoinositide 3 kinase-dependent pathway. The Metformin Combined with Chemotherapy for Pancreatic Cancer Study (NCT01210911) is investigating whether metformin added to erlotinib and gemcitabine enhances the progression-free survival rate in people with locally advanced and metastatic pancreatic cancer 143. The anticancer effect has been proposed because it has been suggested that metformin activates AMPK, which inhibits mammalian target of rapamycin and phosphoinositide 3 kinase that are involved in tumour cell proliferation 45.

### Insulin Analogues

There have been suggestions of a link between insulin analogues and cancer owing to their altered affinity for the insulin receptor 6–150. Four observational studies published in *Diabetologia* examined a possible link between insulin glargine monotherapy and all cancers or breast cancer, with conflicting results 6–151. Further evaluation of these studies confirmed that these data were inconsistent and that the studies in question were subject to a number of important limitations 152–153. Other observational studies have found no connection between insulin glargine use and all cancers 10–13. Owing to the four observational studies in *Diabetologia*, the most recent, large clinical studies have focused on insulin glargine and these are discussed below.

The 6-year, international Outcome Reduction with Initial Glargine Intervention (ORIGIN) Study, designed primarily to determine whether insulin glargine and/or omega-3 fatty acids, could reduce cardiovascular events in 12 537 people with elevated blood glucose levels, had cancer incidence as one of its secondary outcomes. The study compared treatment with insulin glargine with standard care and found, over a median of 6.2 years, no significant difference between study arms in the incidence of all cancers combined (HR: 1.00; 95% CI: 0.88–1.13; p = 0.097); site-specific cancers, including lung cancer (HR: 1.21; 95% CI: 0.87–1.67), colon cancer (HR: 1.09; 95% CI: 0.79–1.51), breast cancer (HR: 1.01, 95% CI: 0.60–1.71), prostate cancer (HR: 0.94; 95% CI: 0.70–1.26), melanoma (HR: 0.88; 95% CI: 0.44–1.75) and other cancers (HR: 0.95; 95% CI: 0.80, 1.14); or cancer mortality (HR: 0.94; 95% CI: 0.77–1.15, p = 0.52) between the insulin glargine and standard of care arms 14. The investigators concluded that insulin glargine had a neutral effect on cancer incidence 14. Other studies have also reported no elevated risk of cancer with insulin glargine compared with other insulins/comparators, such as neutral protamine Hagedorn insulin 154–155, and even a reduced cancer risk with general insulin use 156.

To further investigate the potential link between insulin analogues and cancer, an updated meta-analysis of the Northern European Database Study was performed. This included data from epidemiological studies involving 907 008 subjects. A total of 2 597 602 person-years of observation found no increased risk of developing cancer with insulin glargine versus all other insulins (all cancers combined: summary relative risk: 0.90, 95% CI: 0.82–0.99) 13. There also was no significant increase in the risk of any organ-specific cancer, and the authors concluded that there is no evidence that insulin glargine is associated with an increased risk of cancer compared with all other insulins 13. Another meta-analysis of data from a small population (n = 8693) of people receiving insulin detemir reported no evidence of an increased risk of cancer with insulin detemir compared with neutral protamine Hagedorn insulin (0.36 versus 0.92 events per 100 patient-years' exposure; p < 0.05) and insulin glargine (0.87 versus 1.27; not statistically significant) 157.

Data from two other large database studies (the Kaiser Permanente Collaboration and a US database analysis) were presented at the 2012 American Diabetes Association Scientific Sessions. It was reported that there was no connection between insulin glargine and cancer, and publications on these studies are eagerly awaited 11–12. The International Study of Insulin and Cancer is still ongoing and will provide further information on any insulin-related cancer risk 158. However, a recent study raised issues concerning the use of meta-analyses in determining a link between insulin analogues and cancer 103. It highlighted the fact that current meta-analyses on the subject do not take into account the duration of diabetes or the insulin requirements of the people involved. Both of these variables have implications for the relative distribution of insulin in the liver and periphery, as they affect the amount of insulin being produced in the pancreas and thus the amount required exogenously. Exogenously administered insulin bypasses the first pass through the liver and exposes all tissues to the same dose of insulin, so its clinical role as a potential carcinogenic agent is complex.

### Other Antidiabetic Therapies: Oral

Clinical studies have associated sulfonylureas with an increased risk of cancer 7,8. A retrospective cohort study of 62 809 people with diabetes found that sulfonylureas significantly increased the risk of developing any solid tumour (HR: 1.36; 95% CI: 1.19–1.54; p < 0.001) as well as colorectal (HR: 1.80; 95% CI: 1.29–2.53; p = 0.001) or pancreatic cancer (HR: 4.95; 95% CI: 2.74–8.96; p < 0.001) 8. However, owing to the low number of site-specific cancers reported, the evidence is weak.

*In vitro* and *in vivo* studies on thiazolidinediones have produced inconclusive results; it is possible that these drugs may increase, decrease or have a neutral effect on the risk of cancer or cancer progression 3. Pioglitazone has been shown in a longitudinal study to be weakly associated with an increased risk of bladder cancer after 2 years of use 159. Rosiglitazone was shown to have a neutral effect on the risk of all cancers combined or any common site-specific cancers in a meta-analysis of clinical trials 160. However, the results were inconclusive, owing to the low incidence of site-specific cancers during these trials.

Dipeptidyl peptidase-4 (DPP-4) inhibitors indirectly increase the level of glucagon-like peptide (GLP)-1 in the body by inhibiting its degradation 161. The relationships between DPP-4 and cancer biology are complex, and research on these is ongoing 162. Several studies have found that DPP-4 inhibitors could increase the risk of cancer when used in combination with other agents, including GLP-1 receptor agonists. However, there is currently no evidence that DPP-4 inhibitors increase cancer risk when used alone 163–164.

### Other Antidiabetic Therapies: Injectable

It has been suggested that some GLP-1 based therapies, GLP-1 receptor agonists and DPP-4 inhibitors, could increase the risk of pancreatitis, with a number of cases being reported in the literature. In response to these case reports, analyses have been published in the literature examining the currently available data on incretin drugs. These analyses have reported conflicting results and there is no clear evidence that either DPP-4 inhibitors or GLP-1 receptor agonists are associated with an increased risk for any pancreatic disorder; prospective long-term studies are needed to properly quantify these risks. A number of these analyses are discussed below.

A study investigating GLP-1 based drugs that analysed the US Food and Drug Administration's (FDA) adverse event data reported a sixfold increased risk of pancreatic cancer, as well as an increased risk of thyroid cancer, in people treated with exenatide or sitagliptin (a DPP-4 inhibitor) 164. The FDA, however, has stated that its adverse event data cannot be used to reliably and accurately determine the incidence of an adverse event and conflicting results have been observed in other studies, including preclinical data in animal models 165,166.

In order to address whether sitagliptin use increased the risk for pancreatitis, a pooled analysis including 19 double blind, randomized controlled trials of sitagliptin, which involved 10 246 people with T2DM, was performed, with the analysis adjusted to take into account the different exposures 166. This pooled analysis found that there was no difference between people treated with sitagliptin and controls who had not been exposed to sitagliptin, suggesting that sitagliptin does not increase the risk of pancreatitis. However, this analysis was limited as it did not take other confounding factors that could increase the risk of pancreatitis into account. A meta-analysis has also been conducted examining the GLP-1 receptor agonists exenatide and liraglutide, and whether their use is associated with an increased risk of acute pancreatitis or cancer 168. This included both observational studies (three studies) and randomized controlled trials (22 studies), with 12 studies examining exenatide, 11 studies examining liraglutide and two studies directly comparing the two drugs. The meta-analysis found that neither exenatide [OR: 0.84 (95% CI: 0.58–1.22); *I*^2^ = 30%] or liraglutide [OR: 0.97 (95% CI: 0.21–4.39), *I*^2^ = 0%] was associated with an increased risk of acute pancreatitis. In addition, neither drug was associated with an increased risk of cancer, including thyroid cancer 168.

A retrospective, observational database study compared the risk for acute pancreatitis between the following subpopulations: people with diabetes versus people without diabetes; and between people who had used exenatide, sitagliptin and other antidiabetic therapies 169. This analysis included 38 615 people with diabetes, 6545 exposed to exenatide and 15 826 exposed to sitagliptin, as well as 748 041 people without diabetes. Comparing these groups it was found that the risk of acute pancreatitis was significantly higher in the combined diabetes groups compared with the nondiabetic control groups [adjusted hazard ratio (AHR): 2.1 (95% CI: 1.7–2.5), p < 0.0001]; however there was no difference in risk between people receiving exenatide [AHR: 0.9 (95% CI: 0.6–1.5), p = NS] or sitagliptin [AHR: 0.9 (95% CI: 0.7–1.3), p = NS] compared with other antidiabetic therapies 169. Another large, retrospective database study including 24 237 people initiated on exenatide twice daily, examined the risk of acute pancreatitis compared with people initiated on other antidiabetic medications (n = 457 797) 170. In this analysis inverse weighted propensity scores were used to account for differences in baseline characteristics between the two cohorts, in addition insulin use and the use of any medication known to increase pancreatitis risk were adjusted for. After these adjustments it was found that exposure to exenatide twice daily was not associated with an increased risk for pancreatitis compared with exposure to other antidiabetic medications [OR: 0.95 (95% CI: 0.65–1.38), p = 0.7772]. A secondary analysis that examined whether the risk for pancreatitis was affected by when exenatide exposure had occurred (current, recent or past use), also found that there was no increased risk for any of these exposure times 170. Other large, database studies comparing use of exenatide or sitagliptin in comparison with other antidiabetic agents have found that there is no increased risk of acute pancreatitis with these drugs 171–172.

However, a recent, population-based, case–control study involving 2538 adults with T2DM reported that treatment with exenatide and sitagliptin was associated with increased odds of hospitalization for acute pancreatitis 173. After adjusting for available confounders, current use of GLP-1-based therapies within 30 days [adjusted odds ratio (AOR): 2.24 (95% CI: 1.36−3.69), p = 0.01] and recent use past 30 days and less than 2 years [AOR: 2.01 (95% CI: 1.37−3.18), p = 0.01] were associated with significantly increased odds of acute pancreatitis relative to the odds in nonusers 173. Furthermore, any use was also associated with statistically significant higher odds of acute pancreatitis [AOR: 2.07 (95% CI: 1.36−3.13), p = 0.01] 173. This study must be considered in terms of its limitations, as it was a *post hoc* database study and only a small number of the people included had received exenatide or sitagliptin (34 and 47 of 1269 with pancreatitis, respectively). In addition, a report has suggested that based upon results in mice prone to pancreatic cancer, the use of GLP-1-based therapy may be linked to the progression and transformation of premalignant pancreatic intraepithelial neoplasia 174. An increase in pancreatic mass was observed in people with diabetes mellitus treated with incretin therapy (n = 8) compared with people with diabetes mellitus not treated with incretin therapy (n = 12) 175. This increase in mass was accompanied by both increased exocrine cell proliferation and dysplasia. However, the extremely small sample size and the lack of clarity on whether the controls were qualified to match the cases (controls were age, sex and BMI matched) means that caution should be used when interpreting these results. In addition, this finding has not yet been replicated and firm conclusions cannot therefore be drawn at this time.

Owing to the conflicting results from different trials, the risk of pancreatitis at present can be neither proposed nor excluded. Prospective long-term studies are needed to properly quantify these risks.

Similar concerns have been raised for the GLP-1 receptor agonist liraglutide and the risk for thyroid cancer 174. Liraglutide has been shown to be associated with the development of malignant C-cell carcinoma and thyroid C-cell focal hyperplasia in rats and mice, and as a result, it is recommended that it be prescribed only in cases where the potential benefits are considered to outweigh the risks 176. Liraglutide is currently included in a cancer registry which will monitor its long-term effects over 15 years 177. To date, there is no evidence suggesting that use of liraglutide in humans might be associated with any thyroid pathology.

## Managing the Combined Burden of Diabetes, Obesity and Cancer

Using multidisciplinary treatment strategies that reduce diabetes, obesity and cancer will probably have a greater impact on mortality than tackling each disease individually. Primary prevention should target improvements in lifestyle factors such as smoking cessation and weight management, while patient and physician education on the benefits of these approaches are important. Maintenance of a low waist circumference is a promising way to support cancer prevention and extend life expectancy. Secondary prevention would aim to treat cancer aggressively, manage complications and effectively optimize diabetes management. In general, the American Diabetes Association/American Cancer Society consensus panel recommends that physicians promote a healthy diet, physical activity and weight management for all individuals and routinely screen people with diabetes for cancer. In addition, for the majority of this population, cancer risk should not be a key factor in choosing antidiabetic treatment 3.

## Conclusions

Diabetes, diabetes risk factors and some diabetes treatments may be associated with cancer, with certain cancers developing more commonly in people with T2DM. Strong and plausible evidence exists to suggest links between diabetes, obesity and cancer; however, the underlying mechanisms remain unclear and there is little clinical evidence to guide the appropriate management of people presenting with these diseases concurrently. Joint management and reduction of diabetes, cancer and obesity is likely to result in greater improvements in mortality than treating these diseases separately. Consequently, a multidisciplinary approach is needed to uncover the mechanisms underlying the associations between these diseases and, ultimately, improve clinical outcomes.

## References

[b1] Lopez AD, Mathers CD, Ezzati M, Jamison DT, Murray CJ (2006). Global and regional burden of disease and risk factors, 2001: systematic analysis of population health data. Lancet.

[b2] Jalving M, Gietema JA, Lefrandt JD (2010). Metformin: taking away the candy for cancer?. Eur J Cancer.

[b3] Giovannucci E, Harlan DM, Archer MC (2010). Diabetes and cancer: a consensus report. Diabetes Care.

[b4] Johnson JA, Pollak M (2010). Insulin, glucose and the increased risk of cancer in patients with type 2 diabetes. Diabetologia.

[b5] LeRoith D, Novosyadlyy R, Gallagher EJ, Lann D, Vijayakumar A, Yakar S (2008). Obesity and type 2 diabetes are associated with an increased risk of developing cancer and a worse prognosis; epidemiological and mechanistic evidence. Exp Clin Endocrinol Diabetes.

[b6] Hemkens LG, Grouven U, Bender R (2009). Risk of malignancies in patients with diabetes treated with human insulin or insulin analogues: a cohort study. Diabetologia.

[b7] Bowker SL, Novosyadlyy R, Gallagher EJ, Lann D, Vijayakumar A, Yakar S (2006). Increased cancer-related mortality for patients with type 2 diabetes who use sulfonylureas or insulin. Diabetes Care.

[b8] Currie CJ, Poole CD, Gale EA (2009). The influence of glucose-lowering therapies on cancer risk in type 2 diabetes. Diabetologia.

[b9] Jonasson JM, Ljung R, Talback M, Haglund B, Gudbjornsdottir S, Steineck G (2009). Insulin glargine use and short-term incidence of malignancies-a population-based follow-up study in Sweden. Diabetologia.

[b10] Boyle P (2012). Northern European Database Study of Insulin and Cancer Risk.

[b11] Habel LA (2012). Results from Kaiser-Permanente Collaboration.

[b12] Sturmer T (2012). Cancer link with insulin-data from the U.S. and Northern Europe.

[b13] Boyle P, Koechlin A, Bonio M, Robertson C, Bolli G, Rosenstock J (2012). Updated meta-analysis of cancer risk among users of insulin glargine.

[b14] Gerstein HC, Bosch J, ORIGIN Trial Investigators (2012). Basal insulin and cardiovascular and other outcomes in dysglycemia. N Engl J Med.

[b15] Michels KB, Solomon CG, Hu FB (2003). Type 2 diabetes and subsequent incidence of breast cancer in the Nurses' Health Study. Diabetes Care.

[b16] Hu FB, Manson JE, Liu S (1999). Prospective study of adult onset diabetes mellitus (type 2) and risk of colorectal cancer in women. J Natl Cancer Inst.

[b17] Campbell PT, Deka A, Jacobs EJ (2010). Prospective study reveals associations between colorectal cancer and type 2 diabetes mellitus or insulin use in men. Gastroenterology.

[b18] Friberg E, Mantzoros CS, Wolk A (2007). Diabetes and risk of endometrial cancer: a population-based prospective cohort study. Cancer Epidemiol Biomarkers Prev.

[b19] Parazzini F, La Vecchia C, Negri E (1999). Diabetes and endometrial cancer: an Italian case-control study. Int J Cancer.

[b20] Silverman DT, Schiffman M, Everhart J (1999). Diabetes mellitus, other medical conditions and familial history of cancer as risk factors for pancreatic cancer. Br J Cancer.

[b21] Calle EE, Murphy TK, Rodriguez C, Thun MJ, Heath CWJR (1998). Diabetes mellitus and pancreatic cancer mortality in a prospective cohort of United States adults. Cancer Causes Control.

[b22] Ben Q, Cai Q, Li Z (2011). The relationship between new-onset diabetes mellitus and pancreatic cancer risk: a case-control study. Eur J Cancer.

[b23] Larsson SC, Orsini N, Brismar K, Wolk A (2006). Diabetes mellitus and risk of bladder cancer: a meta-analysis. Diabetologia.

[b24] Larsson SC, Mantzoros CS, Wolk A (2007). Diabetes mellitus and risk of breast cancer: a meta-analysis. Int J Cancer.

[b25] Wolf I, Sadetzki S, Gluck I (2006). Association between diabetes mellitus and adverse characteristics of breast cancer at presentation. Eur J Cancer.

[b26] Larsson SC, Orsini N, Wolk A (2005). Diabetes mellitus and risk of colorectal cancer: a meta-analysis. J Natl Cancer Inst.

[b27] Wolf I, Sadetzki S, Catane R, Karasik A, Kaufman B (2005). Diabetes mellitus and breast cancer. Lancet Oncol.

[b28] Friberg E, Orsini N, Mantzoros CS, Wolk A (2007). Diabetes mellitus and risk of endometrial cancer: a meta-analysis. Diabetologia.

[b29] El-Serag HB, Hampel H, Javadi F (2006). The association between diabetes and hepatocellular carcinoma: a systematic review of epidemiologic evidence. Clin Gastroenterol Hepatol.

[b30] Mitri J, Castillo J, Pittas AG (2008). Diabetes and risk of Non-Hodgkin's lymphoma: a meta-analysis of observational studies. Diabetes Care.

[b31] Chao C, Page JH (2008). Type 2 diabetes mellitus and risk of non-Hodgkin lymphoma: a systematic review and meta-analysis. Am J Epidemiol.

[b32] Everhart J, Wright D (1995). Diabetes mellitus as a risk factor for pancreatic cancer. A meta-analysis. JAMA.

[b33] Huxley R, Ansary-Moghaddam A, Berrington de Gonzalez A, Barzi F, Woodward M (2005). Type-II diabetes and pancreatic cancer: a meta-analysis of 36 studies. Br J Cancer.

[b34] Bonovas S, Filioussi K, Tsantes A (2004). Diabetes mellitus and risk of prostate cancer: a meta-analysis. Diabetologia.

[b35] Kasper JS, Giovannucci E (2006). A meta-analysis of diabetes mellitus and the risk of prostate cancer. Cancer Epidemiol Biomarkers Prev.

[b36] Johnson JA, Carstensen B, Witte D (2012). Diabetes and cancer (1): evaluating the temporal relationship between type 2 diabetes and cancer incidence. Diabetologia.

[b37] Turner EL, Lane JA, Donovan JL (2011). Association of diabetes mellitus with prostate cancer: nested case-control study (Prostate testing for cancer and treatment study). Int J Cancer.

[b38] Bansal D, Bhansali A, Kapil G, Undela K, Tiwari P (2013). Type 2 diabetes and risk of prostate cancer: a meta-analysis of observational studies. Prostate Cancer Prostatic Dis.

[b39] Pierce BL (2012). Why are diabetics at reduced risk for prostate cancer? A review of the epidemiologic evidence. Urol Oncol.

[b40] Liu JH, Li HW, Tong M, Li M, Na YQ (2004). Genetic risk factors of prostate cancer in Han nationality population in Northern China and a preliminary study of the reason of racial difference in prevalence of prostate cancer. Zhonghua Yi Xue Za Zhi.

[b41] Hara N (2012). Prostate carcinogenesis with diabetes and androgen-deprivation-therapy-related diabetes: an update. Exp Diabetes Res.

[b42] Li Q (2010). History of diabetes mellitus and the risk of prostate cancer: the Ohsaki Cohort Study. Cancer Causes Control.

[b43] Tseng CH, Chen CJ, Landolph JR (2012). Diabetes and cancer: epidemiological, clinical, and experimental perspectives. Exp Diabetes Res.

[b44] Al-Delaimy WK, Natarajan L, Rock CL, Sun S, Flatt SW, Pierce JP (2006). Insulin-like growth factor I, insulin-like growth factor I binding protein 1, insulin, glucose, and leptin serum levels are not influenced by a reduced-fat, high-fiber diet intervention. Cancer Epidemiol Biomarkers Prev.

[b45] Stattin P, Bylund A, Rinaldi S (2000). Plasma insulin-like growth factor-I, insulin-like growth factor-binding proteins, and prostate cancer risk: a prospective study. J Natl Cancer Inst.

[b46] Chan JM, Stampfer MJ, Giovannucci E (1998). Plasma insulin-like growth factor-I and prostate cancer risk: a prospective study. Science.

[b47] Nimptsch K, Kenfield S, Jensen MK (2011). Dietary glycemic index, glycemic load, insulin index, fiber and whole-grain intake in relation to risk of prostate cancer. Cancer Causes Control.

[b48] Roddam AW, Allen NE, Appleby P, Key TJ, Endogenous Hormones and Prostate Cancer Collaborative Group (2008). Endogenous sex hormones and prostate cancer: a collaborative analysis of 18 prospective studies. J Natl Cancer Inst.

[b49] Kasper JS, Liu Y, Pollak MN, Rifai N, Giovannucci E (2008). Hormonal profile of diabetic men and the potential link to prostate cancer. Cancer Causes Control.

[b50] Laaksonen DE, Niskanen L, Punnonen K (2004). Testosterone and sex hormone-binding globulin predict the metabolic syndrome and diabetes in middle-aged men. Diabetes Care.

[b51] Pannala R, Leirness JB, Bamlet WR, Basu A, Petersen GM, Chari ST (2008). Prevalence and clinical profile of pancreatic cancer-associated diabetes mellitus. Gastroenterology.

[b52] Ben Q, Xu M, Ning X (2011). Diabetes mellitus and risk of pancreatic cancer: a meta-analysis of cohort studies. Eur J Cancer.

[b53] Huang S, Ye H, Wu W (2013). Research on the relationships between pancreatic cancer and hyperglycemia in Chinese populations. J Diabetes Invest.

[b54] Cui Y, Andersen DK (2012). Diabetes and pancreatic cancer. Endocr Relat Cancer.

[b55] Lipworth L, Zucchetto A, Bosetti C (2011). Diabetes mellitus, other medical conditions and pancreatic cancer: a case-control study. Diabetes Metab Res Rev.

[b56] Grote VA, Rohrmann S, Nieters A (2011). Diabetes mellitus, glycated haemoglobin and C-peptide levels in relation to pancreatic cancer risk: a study within the European Prospective Investigation into Cancer and Nutrition (EPIC) cohort. Diabetologia.

[b57] Pannala R, Basu A, Petersen GM, Chari ST (2009). New-onset diabetes: a potential clue to the early diagnosis of pancreatic cancer. Lancet Oncol.

[b58] Coughlin SS, Calle EE, Teras LR, Petrelli J, Thun MJ (2004). Diabetes mellitus as a predictor of cancer mortality in a large cohort of US adults. Am J Epidemiol.

[b59] Lam EK, Batty GD, Huxley RR (2011). Associations of diabetes mellitus with site-specific cancer mortality in the Asia-Pacific region. Ann Oncol.

[b60] Zhou XH, Qiao Q, Zethelius B (2010). Diabetes, prediabetes and cancer mortality. Diabetologia.

[b61] Barone BB, Yeh HC, Snyder CF (2008). Long-term all-cause mortality in cancer patients with preexisting diabetes mellitus: a systematic review and meta-analysis. JAMA.

[b62] van de Poll-Franse LV, Houterman S, Janssen-Heijnen ML, Dercksen MW, Coebergh JW, Haak HR (2007). Less aggressive treatment and worse overall survival in cancer patients with diabetes: a large population based analysis. Int J Cancer.

[b63] Maruthur NM, Bolen S, Brancati FL, Clark JM (2009). Obesity and mammography: a systematic review and meta-analysis. J Gen Intern Med.

[b64] Pischon T, Boeing H, Hoffmann K (2008). General and abdominal adiposity and risk of death in Europe. N Engl J Med.

[b65] Duggan C, Irwin ML, Xiao L (2011). Associations of insulin resistance and adiponectin with mortality in women with breast cancer. J Clin Oncol.

[b66] Erickson K, Patterson RE, Flatt SW (2011). Clinically defined type 2 diabetes mellitus and prognosis in early-stage breast cancer. J Clin Oncol.

[b67] Lipscombe LL, Fischer HD, Yun L (2012). Association between tamoxifen treatment and diabetes: a population-based study. Cancer.

[b68] Beckman TJ, Cuddihy RM, Scheitel SM, Naessens JM, Killian JM, Pankratz VS (2001). Screening mammogram utilization in women with diabetes. Diabetes Care.

[b69] Richardson LC, Pollack LA (2005). Therapy insight: Influence of type 2 diabetes on the development, treatment and outcomes of cancer. Nat Clin Pract Oncol.

[b70] McBean AM, Yu X (2007). The underuse of screening services among elderly women with diabetes. Diabetes Care.

[b71] Fleming ST, Pursley HG, Newman B, Pavlov D, Chen K (2005). Comorbidity as a predictor of stage of illness for patients with breast cancer. Med Care.

[b72] Maruthur NM, Bolen SD, Brancati FL, Clark JM (2009). The association of obesity and cervical cancer screening: a systematic review and meta-analysis. Obesity (Silver Spring).

[b73] Jiralerspong S, Palla SL, Giordano SH (2009). Metformin and pathologic complete responses to neoadjuvant chemotherapy in diabetic patients with breast cancer. J Clin Oncol.

[b74] Meyerhardt JA, Catalano PJ, Haller DG (2003). Impact of diabetes mellitus on outcomes in patients with colon cancer. J Clin Oncol.

[b75] Weiser MA, Cabanillas ME, Konopleva M (2004). Relation between the duration of remission and hyperglycemia during induction chemotherapy for acute lymphocytic leukemia with a hyperfractionated cyclophosphamide, vincristine, doxorubicin, and dexamethasone/methotrexate-cytarabine regimen. Cancer.

[b76] Barone BB, Yeh HC, Snyder CF (2010). Postoperative mortality in cancer patients with preexisting diabetes: systematic review and meta-analysis. Diabetes Care.

[b77] Perks CM, Vernon EG, Rosendahl AH, Tonge D, Holly JM (2007). IGF-II and IGFBP-2 differentially regulate PTEN in human breast cancer cells. Oncogene.

[b78] Pollak M (2009). Do cancer cells care if their host is hungry?. Cell Metab.

[b79] Gallagher EJ, Fierz Y, Ferguson RD, LeRoith D (2010). The pathway from diabetes and obesity to cancer, on the route to targeted therapy. Endocr Pract.

[b80] Pollak M (2008). Insulin and insulin-like growth factor signalling in neoplasia. Nat Rev Cancer.

[b81] Frasca F, Pandini G, Sciacca L (2008). The role of insulin receptors and IGF-I receptors in cancer and other diseases. Arch Physiol Biochem.

[b82] Papa V, Belfiore A (1996). Insulin receptors in breast cancer: biological and clinical role. J Endocrinol Invest.

[b83] Godsland IF (2010). Insulin resistance and hyperinsulinaemia in the development and progression of cancer. Clin Sci (Lond).

[b84] Ibrahim YH, Yee D (2004). Insulin-like growth factor-I and cancer risk. Growth Horm IGF Res.

[b85] Renehan AG, Frystyk J, Flyvbjerg A (2006). Obesity and cancer risk: the role of the insulin-IGF axis. Trends Endocrinol Metab.

[b86] Frasca F, Pandini G, Scalia P (1999). Insulin receptor isoform A, a newly recognized, high-affinity insulin-like growth factor II receptor in fetal and cancer cells. Mol Cell Biol.

[b87] Weinstein D, Simon M, Yehezkel E, Laron Z, Werner H (2009). Insulin analogues display IGF-I-like mitogenic and anti-apoptotic activities in cultured cancer cells. Diabetes Metab Res Rev.

[b88] Wolf I, Rubinek T, Masur K, Thevenod F, Zanker KS (2008). Diabetes mellitus and breast cancer. Diabetes and cancer.

[b89] Shoelson SE, Lee J, Goldfine AB (2006). Inflammation and insulin resistance. J Clin Invest.

[b90] van Kruijsdijk RC, van der Wall E, Visseren FL (2009). Obesity and cancer: the role of dysfunctional adipose tissue. Cancer Epidemiol Biomarkers Prev.

[b91] Mantovani A, Allavena P, Sica A, Balkwill F (2008). Cancer-related inflammation. Nature.

[b92] Jee SH, Ohrr H, Sull JW, Yun JE, Ji M, Samet JM (2005). Fasting serum glucose level and cancer risk in Korean men and women. JAMA.

[b93] Zeng L, Biernacka KM, Holly JM (2010). Hyperglycaemia confers resistance to chemotherapy on breast cancer cells: the role of fatty acid synthase. Endocr Relat Cancer.

[b94] Villarreal-Garza C, Shaw-Dulin R, Lara-Medina F (2012). Impact of diabetes and hyperglycemia on survival in advanced breast cancer patients. Exp Diabetes Res.

[b95] Turturro F, Friday E, Welbourne T (2007). Hyperglycemia regulates thioredoxin-ROS activity through induction of thioredoxin-interacting protein (TXNIP) in metastatic breast cancer-derived cells MDA-MB-231. BMC Cancer.

[b96] Pandey V, Chaube B, Bhat MK (2011). Hyperglycemia regulates MDR-1, drug accumulation and ROS levels causing increased toxicity of carboplatin and 5-fluorouracil in MCF-7 cells. J Cell Biochem.

[b97] Sadoff L (1998). Overwhelming 5-fluorouracil toxicity in patients whose diabetes is poorly controlled. Am J Clin Oncol.

[b98] Brunello A, Kapoor R, Extermann M (2011). Hyperglycemia during chemotherapy for hematologic and solid tumors is correlated with increased toxicity. Am J Clin Oncol.

[b99] Kung SS, Goldberg ND, Dahl JL, Parks REJR, Kline BE (1963). Potentiation of 5-Fluorouracil Inhibition of Flexner-Jobling Carcinoma by Glucose. Science.

[b100] Feng J-P, Yuan X-L, Li M (2012). Secondary diabetes associated with 5-fluorouracil-based chemotherapy regimens in non-diabetic patients with colorectal cancer: results from a single-centre cohort study. Colorectal Dis.

[b101] Clore JN, Thurby-Hay L (2009). Glucocorticoid-induced hyperglycemia. Endocr Pract.

[b102] Pennell NA (2012). Selection of chemotherapy for patients with advanced non-small cell lung cancer. Cleve Clin J Med.

[b103] Vigneri P, Frasca F, Sciacca L, Pandini G, Vigneri R (2009). Diabetes and cancer. Endocr Relat Cancer.

[b104] Feller S, Boeing H, Pischon T (2010). Body mass index, waist circumference, and the risk of type 2 diabetes mellitus: implications for routine clinical practice. Dtsch Arztebl Int.

[b105] Pischon T, Boeing H, Weikert S (2008). Body size and risk of prostate cancer in the European prospective investigation into cancer and nutrition. Cancer Epidemiol Biomarkers Prev.

[b106] Berrington de Gonzalez A, Spencer EA, Bueno-de-Mesquita HB (2006). Anthropometry, physical activity, and the risk of pancreatic cancer in the European prospective investigation into cancer and nutrition. Cancer Epidemiol Biomarkers Prev.

[b107] Calle EE, Rodriguez C, Walker-Thurmond K, Thun MJ (2003). Overweight, obesity, and mortality from cancer in a prospectively studied cohort of U.S. adults. N Engl J Med.

[b108] Renehan AG, Tyson M, Egger M, Heller RF, Zwahlen M (2008). Body-mass index and incidence of cancer: a systematic review and meta-analysis of prospective observational studies. Lancet.

[b109] Wolin KY, Carson K, Colditz GA (2010). Obesity and cancer. Oncologist.

[b110] Steffen A, Schulze MB, Pischon T (2009). Anthropometry and esophageal cancer risk in the European prospective investigation into cancer and nutrition. Cancer Epidemiol Biomarkers Prev.

[b111] Friedenreich C, Norat T, Steindorf K (2006). Physical activity and risk of colon and rectal cancers: the European prospective investigation into cancer and nutrition. Cancer Epidemiol Biomarkers Prev.

[b112] Pischon T, Lahmann PH, Boeing H (2006). Body size and risk of colon and rectal cancer in the European Prospective Investigation Into Cancer and Nutrition (EPIC). J Natl Cancer Inst.

[b113] Lahmann PH, Hoffmann K, Allen N (2004). Body size and breast cancer risk: findings from the European Prospective Investigation into Cancer And Nutrition (EPIC). Int J Cancer.

[b114] Vucenik I, Stains JP (2012). Obesity and cancer risk: evidence, mechanisms, and recommendations. Ann N Y Acad Sci.

[b115] Griggs JJ, Sorbero ME, Lyman GH (2005). Undertreatment of obese women receiving breast cancer chemotherapy. Arch Intern Med.

[b116] Wong JR, Gao Z, Merrick S (2009). Potential for higher treatment failure in obese patients: correlation of elevated body mass index and increased daily prostate deviations from the radiation beam isocenters in an analysis of 1,465 computed tomographic images. Int J Radiat Oncol Biol Phys.

[b117] Calle EE, Kaaks R (2004). Overweight, obesity and cancer: epidemiological evidence and proposed mechanisms. Nat Rev Cancer.

[b118] Landman GW, Van Hateren KJ, Kleefstra N, Bilo HJ (2010). The relationship between obesity and cancer mortality in type 2 diabetes: a ten-year follow-up study (ZODIAC-21). Anticancer Res.

[b119] Williams GP (2010). The role of oestrogen in the pathogenesis of obesity, type 2 diabetes, breast cancer and prostate disease. Eur J Cancer Prev.

[b120] Alokail MS, Al-Daghri NM, Al-Attas OS, Hussain T (2009). Combined effects of obesity and type 2 diabetes contribute to increased breast cancer risk in premenopausal women. Cardiovasc Diabetol.

[b121] Ali AS, Ali S, Ahmad A, Bao B, Philip PA, Sarkar FH (2011). Expression of microRNAs: potential molecular link between obesity, diabetes and cancer. Obes Rev.

[b122] Percik R, Stumvoll M (2009). Obesity and cancer. Exp Clin Endocrinol Diabetes.

[b123] Stattin P, Lukanova A, Biessy C (2004). Obesity and colon cancer: does leptin provide a link?. Int J Cancer.

[b124] Fenton JI, Hord NG, Lavigne JA, Perkins SN, Hursting SD (2005). Leptin, insulin-like growth factor-1, and insulin-like growth factor-2 are mitogens in ApcMin/+ but not Apc+/+ colonic epithelial cell lines. Cancer Epidemiol Biomarkers Prev.

[b125] Wellen KE, Thompson CB (2010). Cellular metabolic stress: considering how cells respond to nutrient excess. Mol Cell.

[b126] Bao B, Wang Z, Li Y (2011). The complexities of obesity and diabetes with the development and progression of pancreatic cancer. Biochim Biophys Acta.

[b127] Ramos EJ, Xu Y, Romanova I (2003). Is obesity an inflammatory disease?. Surgery.

[b128] Fogarty AW, Glancy C, Jones S, Lewis SA, McKeever TM, Britton JR (2008). A prospective study of weight change and systemic inflammation over 9 y. Am J Clin Nutr.

[b129] Tsujinaka S, Konishi F, Kawamura YJ (2008). Visceral obesity predicts surgical outcomes after laparoscopic colectomy for sigmoid colon cancer. Dis Colon Rectum.

[b130] Balentine CJ, Wilks J, Robinson C (2010). Obesity increases wound complications in rectal cancer surgery. J Surg Res.

[b131] Makino T, Shukla PJ, Rubino F, Milsom JW (2012). The impact of obesity on perioperative outcomes after laparoscopic colorectal resection. Ann Surg.

[b132] Park JW, Lim SW, Choi HS, Jeong SY, Oh JH, Lim SB (2010). The impact of obesity on outcomes of laparoscopic surgery for colorectal cancer in Asians. Surg Endosc.

[b133] Whitlock K, Gill RS, Birch DW, Karmali S (2012). The association between obesity and colorectal cancer. Gastroenterol Res Pract.

[b134] Griggs JJ, Mangu PB, Anderson H (2012). Appropriate chemotherapy dosing for obese adult patients with cancer: American Society of Clinical Oncology clinical practice guideline. J Clin Oncol.

[b135] Zakikhani M, Dowling R, Fantus IG, Sonenberg N, Pollak M (2006). Metformin is an AMP kinase-dependent growth inhibitor for breast cancer cells. Cancer Res.

[b136] Dowling RJ, Zakikhani M, Fantus IG, Pollak M, Sonenberg N (2007). Metformin inhibits mammalian target of rapamycin-dependent translation initiation in breast cancer cells. Cancer Res.

[b137] Burton JD, Goldenberg DM, Blumenthal RD (2008). Potential of peroxisome proliferator-activated receptor gamma antagonist compounds as therapeutic agents for a wide range of cancer types. PPAR Res.

[b138] Tachibana K, Yamasaki D, Ishimoto K, Doi T (2008). The role of PPARs in cancer. PPAR Res.

[b139] Nathan DM, Buse JB, Davidson MB (2009). Medical management of hyperglycemia in type 2 diabetes: a consensus algorithm for the initiation and adjustment of therapy: a consensus statement of the American Diabetes Association and the European Association for the Study of Diabetes. Diabetes Care.

[b140] Li D, Yeung SC, Hassan MM, Konopleva M, Abbruzzese JL (2009). Antidiabetic therapies affect risk of pancreatic cancer. Gastroenterology.

[b141] Bowker SL, Yasui Y, Veugelers P, Johnson JA (2010). Glucose-lowering agents and cancer mortality rates in type 2 diabetes: assessing effects of time-varying exposure. Diabetologia.

[b142] Evans JM, Donnelly LA, Emslie-Smith AM, Alessi DR, Morris AD (2005). Metformin and reduced risk of cancer in diabetic patients. BMJ.

[b143] Libby G, Donnelly LA, Donnan PT, Alessi DR, Morris AD, Evans JM (2009). New users of metformin are at low risk of incident cancer: a cohort study among people with type 2 diabetes. Diabetes Care.

[b144] Kourelis TV, Siegel RD (2012). Metformin and cancer: new applications for an old drug. Med Oncol.

[b145] Wilmik J http://clinicaltrials.gov/ct2/show/NCT01210911.

[b146] Stevens R, Ali R, Bankhead CR (2012). Cancer outcomes and all-cause mortality in adults allocated to metformin: systematic review and collaborative meta-analysis of randomised clinical trials. Diabetologia.

[b147] Bosco JL, Antonsen S, Sorensen HT, Pedersen L, Lash TL (2011). Metformin and incident breast cancer among diabetic women: a population-based case-control study in Denmark. Cancer Epidemiol Biomarkers Prev.

[b148] Little MW, Pugh TF, Carey FJ (2011). The potential protective effect of metformin against pancreatic cancer: preliminary results from a case–control study in two UK centres. Gut.

[b149] Mannucci E, Monami M, Balzi D (2010). Doses of insulin and its analogues and cancer occurrence in insulin-treated type 2 diabetic patients. Diabetes Care.

[b150] Yang YX, Hennessy S, Lewis JD (2004). Insulin therapy and colorectal cancer risk among type 2 diabetes mellitus patients. Gastroenterology.

[b151] Colhoun HM, SDRN Epidemiology Group (2009). Use of insulin glargine and cancer incidence in Scotland: a study from the Scottish Diabetes Research Network Epidemiology Group. Diabetologia.

[b152] Pocock SJ, Smeeth L (2009). Insulin glargine and malignancy: an unwarranted alarm. Lancet.

[b153] Smith U, Gale EA (2009). Does diabetes therapy influence the risk of cancer?. Diabetologia.

[b154] Rosenstock J, Fonseca V, McGill JB (2009). Similar risk of malignancy with insulin glargine and neutral protamine Hagedorn (NPH) insulin in patients with type 2 diabetes: findings from a 5 year randomised, open-label study. Diabetologia.

[b155] Home PD, Lagarenne P (2009). Combined randomised controlled trial experience of malignancies in studies using insulin glargine. Diabetologia.

[b156] Yang X, Ko GT, So WY (2010). Associations of hyperglycemia and insulin usage with the risk of cancer in type 2 diabetes: the Hong Kong diabetes registry. Diabetes.

[b157] Dejgaard A, Lynggaard H, Rastam J, Krogsgaard Thomsen M (2009). No evidence of increased risk of malignancies in patients with diabetes treated with insulin detemir: a meta-analysis. Diabetologia.

[b158] Grimaldi-Bensouda L, Marty M, Pollak M (2010). The international study of insulin and cancer. Lancet.

[b159] Lewis JD, Ferrara A, Peng T (2011). Risk of bladder cancer among diabetic patients treated with pioglitazone: interim report of a longitudinal cohort study. Diabetes Care.

[b160] Monami M, Lamanna C, Marchionni N, Mannucci E (2008). Rosiglitazone and risk of cancer: a meta-analysis of randomized clinical trials. Diabetes Care.

[b161] McIntosh CH, Demuth HU, Pospisilik JA, Pederson R (2005). Dipeptidyl peptidase IV inhibitors: how do they work as new antidiabetic agents?. Regul Pept.

[b162] Stulc T, Sedo A (2010). Inhibition of multifunctional dipeptidyl peptidase-IV: is there a risk of oncological and immunological adverse effects?. Diabetes Res Clin Pract.

[b163] Masur K, Schwartz F, Entschladen F, Niggemann B, Zaenker KS (2006). DPPIV inhibitors extend GLP-2 mediated tumour promoting effects on intestinal cancer cells. Regul Pept.

[b164] Elashoff M, Matveyenko AV, Gier B, Elashoff R, Butler PC (2011). Pancreatitis, pancreatic, and thyroid cancer with glucagon-like peptide-1-based therapies. Gastroenterology.

[b165] US Food and Drug Administration (2010). FDA Adverse Event Reporting System (FAERS) (formerly AERS). http://www.fda.gov/drugs/guidancecomplianceregulatoryinformation/surveillance/adversedrugeffects/default.htm.

[b166] Engel SS, Williams-Herman DE, Golm GT (2010). Sitagliptin: review of preclinical and clinical data regarding incidence of pancreatitis. Int J Clin Pract.

[b167] Tatarkiewicz K, Smith PA, Sablan EJ (2010). Exenatide does not evoke pancreatitis and attenuates chemically induced pancreatitis in normal and diabetic rodents. Am J Physiol Endocrinol Metab.

[b168] Alves C, Batel-Marques F, Macedo AF A meta-analysis of serious adverse events reported with exenatide and liraglutide: acute pancreatitis and cancer. Diabetes Res Clin Pract.

[b169] Garg R, Chen W, Pendergrass M (2012–2010). Acute pancreatitis in type 2 diabetes treated with exenatide or sitagliptin: a retrospective observational pharmacy claims analysis. Diabetes Care.

[b170] Wenten M, Gaebler JA, Hussein M (2012). Relative risk of acute pancreatitis in initiators of exenatide twice daily compared with other anti-diabetic medication: a follow-up study. Diabet Med.

[b171] Dore DD, Bloomgren GL, Wenten M (2011). A cohort study of acute pancreatitis in relation to exenatide use. Diabetes Obes Metab.

[b172] Dore DD, Seeger JD, Arnold Chan K (2009). Use of a claims-based active drug safety surveillance system to assess the risk of acute pancreatitis with exenatide or sitagliptin compared to metformin or glyburide. Curr Med Res Opin.

[b173] Singh S, Chang HY, Richards TM, Weiner JP, Clark JM, Segal JB (2013). Glucagonlike peptide 1-based therapies and risk of hospitalization for acute pancreatitis in type 2 diabetes mellitus: a population-based matched case-control study.

[b174] Gier B, Butler PC Glucagon like peptide 1-based drugs and pancreatitis: clarity at last, but what about pancreatic cancer?. JAMA Intern Med.

[b175] Butler AE, Campbell-Thompson M, Gurlo T, Dawson DW, Atkinson M, Butler PC (2013). Marked expansion of exocrine and endocrine pancreas with incretin therapy in humans with increased exocrine pancreas dysplasia and the potential for glucagon-producing neuroendocrine tumors. Diabetes.

[b176] Novo Nordisk. (2011). Victoza® (liraglutide [rDNA origin] injection)solution for subcutaneous use: US prescribing information.

[b177] Parks M, Rosebraugh C (2010). Weighing risks and benefits of liraglutide--the FDA's review of a new antidiabetic therapy. N Engl J Med.

